# Expressions of IGF-1R and Ki-67 in breast cancer patients with diabetes mellitus and an analysis of biological characteristics

**DOI:** 10.12669/pjms.38.1.4718

**Published:** 2022

**Authors:** Xiaolu Yan, Zhe Gao, Ye Zhou, Fei Gao, Qingxia Li

**Affiliations:** 1 Xiaolu Yan, Department of Oncology, Hebei General Hospital, Shijiazhuang 050051, Hebei, China; 2 Zhe Gao, Department of Endocrinology and Metabolic Diseases, Hebei General Hospital, Shijiazhuang 050051, Hebei, China; 3 Ye Zhou, Department of Oncology, Hebei General Hospital, Shijiazhuang 050051, Hebei, China; 4 Fei Gao, Department of Oncology, Hebei General Hospital, Shijiazhuang 050051, Hebei, China; 5 Qingxia Li, Department of Oncology, Hebei General Hospital, Shijiazhuang 050051, Hebei, China

**Keywords:** Type-2 diabetes mellitus, Breast cancer, Insulin-like growth factor-1 receptor, Proliferating cell nuclear antigen, Prognosis

## Abstract

**Objectives:**

There is a cross-link of insulin and insulin-like growth factor-1 (IGF-1) with each other’s receptors. The present study was carried out to explore the relationship of Type-2 diabetes mellitus (T2DM) with the occurrence and development of breast cancer by analyzing the expression of IGF-1R and Ki-67, as well as the biological characteristics in breast cancer patients with and without diabetes mellitus.

**Methods:**

A total of 102 cases of breast cancer patients with T2DM admitted in Hebei General Hospital from January 2019 to December 2020 were selected and grouped in T2DM group. While the control group included 106 cases of breast cancer patients without diabetes mellitus in the same period. Further comparison was conducted focusing on the general data, clinical stage, tumor histological grade, molecular classification and prognosis, and the expressions of IGF-1R and Ki-67 in breast cancer tissue between groups.

**Results:**

Compared with control group, patients in T2DM group were elderly and accounted for a larger proportion of post-menopause (*p*<0.05), yet with no significant difference in body mass and family history (*p*>0.05). Compared with control group, T2DM group had advanced clinical stage, higher histological grade, and more common molecular type, with statistical differences between groups (*p*<0.05). Furthermore, there were higher proportions of local recurrence, lymph node metastasis and distant metastasis in T2DM group than those in control group, yet with no statistical significance (*p*>0.05). While statistical difference was found in the comparison of the 5-year survival rate, which was lower in T2DM group than that in control group (*p*<0.05). In addition, compared with control group, there were significant increase in both the expressions of IGF-1R and Ki-67 in T2DM group (*p*<0.05).

**Conclusions:**

T2DM may be one of the risk factors affecting the occurrence, development and prognosis of breast cancer, which may decrease the 5-year survival of breast cancer patients. Besides, high expressions of IGF-1R and Ki-67 may be the key factors for poor prognosis of breast cancer patients with diabetes mellitus.

## INTRODUCTION

Breast cancer ranks the first (about 28%) in female malignant tumors, which seriously endangers women’s life and health.^1^,^2^ Simultaneously, Type-2 diabetes mellitus (T2DM) has also become a major global public health problem. It has been predicted that the total number of adult patients with T2DM will increase from 170 million to 360 million from 2000 to 2030 globally.^3^ Furthermore, prior studies support that diabetes mellitus is one of the high-risk factors of breast cancer, 20% of such group of patients in the early stage have diabetes mellitus, and its related mechanisms include hyperglycemia, hyperinsulinemia, etc.^4^,^5^ Meanwhile, there is a cross-link of insulin and insulin-like growth factor-1 (IGF-1) with each other’s receptors. Insulin-like growth factor-1 receptor (IGF-1R), a transmembrane tyrosine protein receptor, is highly expressed in nervous system malignancies, hepatocellular carcinoma, breast cancer, adrenocortical tumors and lung cancer. Besides, it plays an important role in cell proliferation, differentiation and apoptosis by binding with its ligands. Ki-67 protein is a tumor proliferation antigen that is related to cell cycle. It can effectively evaluate the proliferative activity of tumor cells,^6^ which is associated with the occurrence, development and prognosis of various tumors. In view of the above, the present study retrospectively analyzed the clinicopathological characteristics and the expressions of IGF-1R and Ki-67 in breast cancer tissues of patients with and without diabetes mellitus in our hospital. The purpose of our study was to explore the effect of T2DM on the biological characteristics of breast cancer, and to investigate whether the difference of IGF-1R and Ki-67 expressions in breast cancer affects the prognosis of these patients with diabetes mellitus. It is expected to provide experimental and theoretical basis for evaluating the relationship of diabetes mellitus with the occurrence and development of breast cancer, which is of great significance for the prevention and treatment of breast cancer.

## METHODS

### Ethical Approval:

The study was approved by the Institutional Ethics Committee of Hebei General Hospital on July 08, 2019(No.K20190423), and written informed consent was obtained from all participants.

### Inclusion Criteria:

A total of 102 cases of breast cancer patients with T2DM admitted in Hebei General Hospital hospital from January 2019 to December 2020 were selected and grouped in T2DM group. The diagnostic criteria of diabetes mellitus referred to the diagnostic criteria proposed by World Health Organization/International Diabetes Federation (WHO/IDF) in 1999. While the control group included 106 cases of breast cancer patients without diabetes mellitus in the same period. All the enrolled patients were treated by surgery and confirmed as invasive breast cancer by postoperative pathological examination.

### Exclusion Criteria:

Patients with Type-2 diabetes mellitus, secondary diabetes mellitus, breast cancer in situ and bilateral breast cancer, as well as male breast cancer patients.

After data sorting of enrolled patients in the two groups, a comparison was made focusing on the general data (age, weight, family history, menopausal status), clinical stage, tumor histological grade, molecular typing, lymph node and distant metastasis, and 5-year survival rate, followed by the detection of breast cancer tissue. The clinicopathological staging was performed according to the American Joint Committee on Cancer (AJCC): the 7th Edition of the AJCC Cancer Staging Manual. The histological grading was conducted based on modified Scarff-Bloom-Richardon (SBR) grading system. In addition, molecular typing of breast cancer referred to St. Gallen International Breast Cancer Conference in March 2011. The blood glucose control was strengthened before and after operation, and a 5-year follow-up was carried out according to the follow-up principle of breast cancer.

### Detection of IGF-1R and Ki-67 expressions:

The expressions of IGF-1R and Ki-67 in breast cancer tissues were detected by immunohistochemistry of streptomycin avidin-biotin-peroxidase complex (S-P) method in accordance with the instructions specifically. In terms of result determination, IGF-1R was positive when stained in pale yellow and brownish yellow in the membrane, and Ki-67 positive cells were brown and tan granules with clear nuclear boundary. The modified Sinicrope method was used to determine the staining results of both IGF-1R and Ki-67. Percentage of positive cells: Stained cells <5%, 0 point; stained cells of 6%~25%, 1 point; stained cells of 26%~50%, 2 points; stained cells of 51%~75%, 3 points; and stained cells >75%, 4 points. The staining intensity was divided into 4 grades: 0 point: no color; 1 point: pale yellow; 2 points: dark brownish yellow; and 3 point: brownish black. Scoring per section was realized by multiplying the counting score of stained cells by the score of staining intensity. Finally, the results were determined based on the obtained scores as follows: 0~1 point, negative (-); 2~3 points, weak positive (+), 4~6 points, moderate positive (++), ≥7 points, strong positive (+++), of which negative expression was based on (-) and (+), while (++) and (+++) were positive.^7^

### Statistical Analysis:

SPSS18.0 statistical software was used for statistical analysis in this study. Measurement data were expressed as mean ± standard deviation and compared using *t*-test, and the counting data were processed by *χ*^2^ test. *P*<0.05 showed that the difference was statistically significant.

## RESULTS

There was no significant difference in body mass and family history between T2DM group and control group (*p*>0.05). In addition, compared with control group, patients in T2DM group were elder and accounted for a larger proportion of post-menopause (*p*<0.05). (Table-I). In comparison to the control group, T2DM group had advanced clinical stage, higher histological grade, and more common molecular type, with statistical differences between groups (*p*<0.05), suggesting a poorer prognosis in T2DM group. (Table-II).

**Table-I:** Comparison of general data between T2DM group and control group [x̄±s, n (%)].

Groups	Cases	Age (years)	Body mass (Kg/m²)	Family history [n (%)]	Menopausal status [n (%)] Ing Pre-menopause Post-menopause	
T2DM group	102	56.26±8.69	27.35±3.28	20	33(32.4)	69(67.6)
Control group	106	50.48±9.16	26.85±3.42	18	46(43.4)	60(56.6)

**Table-II:** Clinical staging, histological grading and molecular typing of T2DM group and control group [n (%)].

		Clinical staging				Histological grading			Molecular typing			

Groups	Cases	I	II	III	IV	1	2	3	Luminal A	Luminal B	ERBB2+	Basal-like
T2DM group	102	26 (25.5)	39 (38.2)	37 (36.3)	0 (0)	60 (58.9)	24 (23.5)	18 (17.6)	21 (20.6)	38 (37.3)	8 (7.8)	35 (34.3)
Control group	106	47 (44.3)	34 (32.1)	25 (23.6)	0 (0)	65 (61.3)	32 (30.2)	9 (8.5)	14 (13.2)	54 (51.0)	13 (12.2)	25 (23.6)

Compared with control group, there were higher proportions of local recurrence, lymph node metastasis and distant metastasis in T2DM group, yet with no statistical significance (*p*>0.05). While statistical difference was found in the comparison of the 5-year survival rate, which was lower in T2DM group than that in control group (*p*<0.05). (Table-III).

**Table-III:** Comparison of prognosis between T2DM group and control group.

Groups	Cases	Local recurrence (n)	Lymph node metastasis (n)	Distant metastasis (n)	5-year survival rate (%)
T2DM group	102	10	14	17	77.5%
Control group	106	7	11	12	85.8%

Compared with control group, there were significant increase in both the expressions of IGF-1R and Ki-67 in T2DM group (*p*<0.05). (Table-IV).

**Table-IV:** Comparison of IGF-1R and Ki-67 expressions between T2DM group and control group [n (%)].

Groups	Cases (n)	IGF-1R		Ki-67	

		Positive	Negative	Positive	Negative
T2DM group	102	62(60.8)	40(39.2)	57(55.9)	45(44.1)
Control group	108	53(49.1)	55(50.9)	45(41.7)	63(58.3)

## DISCUSSION

With the development of modern society, malignant tumors and diabetes mellitus have become the two common diseases threatening human health and life, both of which have gradual increasing morbidity and mortality year after year. Both domestic and foreign studies report an increased incidence of malignant tumors in patients with diabetes mellitus. The morbidity and mortality of breast cancer are the highest in female malignancies.^1^,^8^ The relationship between diabetes mellitus and breast cancer has been a matter of great concern. T2DM may be one of the high-risk factors of breast cancer.^9^ Considering the heterogeneity of breast cancer, its varied clinical characteristics and molecular gene phenotypes may produce different treatment response and prognosis.^10^ Accordingly, the present study analyzed the clinical characteristics and molecular gene phenotype differences of breast cancer patients with T2DM, the expressions of IGF-1R and Ki-67, and their impact on patient prognosis.

In our study, there was no significant difference in body mass index between the two groups, indicating that obesity may be a risk factor for both diabetes mellitus and breast cancer. Previous evidence-based medicine confirmed that overweight and obesity can increase the risk of postmenopausal breast cancer.^11^ Its pathogenesis can be explained by the reason that obesity can elevate the count of adipocytokines and change the levels and biological activities of estrogen, insulin, leptin and other hormones, and the status of chronic inflammation.^12^ For instance, Li reported in their research that breast cancer patients with T2DM were elder and accounted for a larger proportion of entering menopause^13^; besides, Michels discovered that the risk of breast cancer in patients with T2DM was 17% higher than that in non-diabetic patients, especially in postmenopausal women.^14^ Similarly, our study revealed that patients with T2DM were elderly, and the proportion of postmenopausal women was 67.6% in this group, significantly higher than that in the control group (56.7%).

As regards clinical stage, histological grade and molecular typing, Peairs^15^ reported that breast cancer patients with T2DM had advanced clinical stage and poorer prognosis. In the current study, the clinical stage of T2DM group was later than that of the control group, and the proportion of patients at stage II/III in the former group (74.5%) was significantly higher than that of the latter group (55.7%), which was consistent with the previous research. Furthermore, there is also difference in the prognosis of breast cancer patients with different molecular types, with the worst outcome in patients with triple negative breast cancer. Findings of our study revealed that patients in T2DM group had higher histological grade and accounted for a higher proportion of basal-like type (triple negative breast cancer), similar to those reported in the literature.^15^ At the same time, according to the analysis of the prognosis of breast cancer patients with T2DM, the local recurrence, lymph node metastasis and distant metastasis ratio of patients in T2DM group were higher than those in the control group, yet with the absence of statistical difference, with a decreased 5-year survival rate in T2DM group, however. Similarly, Villareal^16^ carried out a prospective analysis on patients with recurrent and metastatic breast cancer, and found that the overall survival time of hyperglycemia group (>130 mg/dl) was significantly shorter than that of normal glucose group. In addition, Erickson^17^ also showed in their research a worse prognosis of female breast cancer patients with HbA1c >7%, and the overall survival rate was lower than that of the control group.

In order to further clarify the relationship of diabetes mellitus with the occurrence and development of breast cancer, the expressions of IGF-1R and Ki-67 were detected in breast cancer tissues of patients with/without diabetes mellitus. Ki-67 antigen is a protein related to cell division and proliferation. It has now been recognized to be a proliferation marker gene, which is related to the occurrence, development, metastasis and prognosis of various tumors.^18^ Owing to a short half-life of Ki-67, it has become a reliable indicator of tumor cell proliferation. For example, Yamashita^19^ reported a positive correlation of Ki-67 expression with the histological grade in breast cancer. In addition, prior research has documented that Ki-67 showed a higher expression in breast cancer with larger tumor size, and was associated with the clinical stage of this cancer. In this study, compared with control group, T2DM group had higher positive rate of Ki-67, advanced clinical stage and higher histological grade. These results suggest that abnormal glucose metabolism may further promote the development of breast cancer. IGF-1, encoded by a gene on chromosome 12, is a 70 amino acid basic single-chain polypeptide which displays relatively-high homology (60%) with insulin. Due to the existence of insulin resistance in T2DM, IGF-1 and insulin are at a high level over a long period of time, with secondary elevation in IGF-1R. Importantly, IGF-1 plays a biological role mainly through IGF-1R.^20^ Subsequently, IGF-1 binds to IGF-1R to phosphorylate and inactivate BAD protein in Bcl family through the activation of phosphoinositide 3-kinase (PI3K)/Akt or Ras/Raf/mitogen activated protein kinase (MAPK) signaling pathway, hence preventing cells from apoptosis.^21^ Simultaneously, MAPK pathway can also transfer signals to the cell nucleus to induce the activation of nuclear transcription factors and promote cell proliferation, exhibiting a direct association with the occurrence and development of tumors.^22^ As revealed in our study, there was an increased positive rate of IGF-1R in T2DM group than that in control group. Meanwhile, in accordance with the analysis results of the biological characteristics and prognosis of breast cancer patients with T2DM, T2DM group displayed advanced clinical stage, higher histological grade and higher proportion of basic-like type (i.e. three negative breast cancer), and reduced 5-year survival rate. These findings suggest that the occurrence and development of breast cancer may be promoted owing to the abnormal glucose metabolism and secondary increase of IGF-1R in patients with diabetes mellitus. Similarly, Christopoulos also demonstrated an important role of the IGF-1 system and its receptors in the occurrence, progression and metastasis of breast cancer.^23^

### Limitations and Recommendations of the study:

Nevertheless, deficiencies are still visible in this study: small sample size, short follow-up time, and no more accurate classification study on T2DM patients. In addition, only patients with mild to moderate T2DM are selected as subjects to ensure the effectiveness of the study. Based on this, relevant countermeasures are being carried out to actively enrich the sample content, further extend the time of follow-up, and classify different types and further include patients with severe T2DM in the study, so as to conduct a more objective evaluation of the efficacy of this treatment regimen for patients with different severity and disparate types of T2DM.

## CONCLUSIONS

T2DM may be one of the risk factors affecting the occurrence, development and prognosis of breast cancer, which may decrease the 5-year survival of breast cancer patients. Besides, high expressions of IGF-1R and Ki-67 may be essential factors for poor prognosis of breast cancer patients with diabetes mellitus. Findings in our study suggest that further intervention of the intermediate links may be feasible during the treatment of breast cancer patients with T2DM, which provide theoretical basis and new insights for the prevention and treatment of breast cancer.

### Authors’ Contributions:

**XY & ZG:** Designed this study and prepared this manuscript, and are responsible and accountable for the accuracy or integrity of the work.

**YZ & FG:** Collected and analyzed clinical data.

**QL** significantly revised this manuscript.
